# Functional Use of Directional Local Field Potentials in the Subthalamic Nucleus Deep Brain Stimulation

**DOI:** 10.3389/fnhum.2020.00145

**Published:** 2020-04-28

**Authors:** Ilknur Telkes, Shelby Sabourin, Jennifer Durphy, Octavian Adam, Vishad Sukul, Nataly Raviv, Michael D. Staudt, Julie G. Pilitsis

**Affiliations:** ^1^Department of Neuroscience and Experimental Therapeutics, Albany Medical College, Albany, NY, United States; ^2^Department of Neurology, Albany Medical Center, Albany, NY, United States; ^3^Department of Neurosurgery, Albany Medical Center, Albany, NY, United States

**Keywords:** Parkinson’s disease, directional leads, local field potentials, subthalamic nucleus, spectral analysis

## Abstract

**Background:**

Directional deep brain stimulation (DBS) technology aims to address the limitations, such as stimulation-induced side effects, by delivering selective, focal modulation via segmented contacts. However, DBS programming becomes more complex and time-consuming for clinical feasibility. Local field potentials (LFPs) might serve a functional role in guiding clinical programming.

**Objective:**

In this pilot study, we investigated the spectral dynamics of directional LFPs in subthalamic nucleus (STN) and their relationship to motor symptoms of Parkinson’s disease (PD).

**Methods:**

We recorded intraoperative STN-LFPs from 8-contact leads (Infinity-6172, Abbott Laboratories, Illinois, United States) in 8 PD patients at rest. Directional LFPs were referenced to their common average and time-frequency analysis was computed using a modified Welch periodogram method. The beta band (13–35 Hz) features were extracted and their correlation to preoperative UPDRS-III scores were assessed.

**Results:**

Normalized beta power (13–20 Hz) and normalized peak power (13–35 Hz) were found to be higher in anterior direction despite lack of statistical significance (*p* > 0.05). Results of the Spearman correlation analysis demonstrated positive trends with bradykinesia/rigidity in dorsoanterior direction (*r* = 0.659, *p* = 0.087) and with axial scores in the dorsomedial direction (*r* = 0.812, *p* = 0.072).

**Conclusion:**

Given that testing all possible combinations of contact pairs and stimulation parameters is not feasible in a single clinic visit, spatio-spectral LFP dynamics obtained from intraoperative recordings might be used as an initial marker to select optimal contact(s).

## Introduction

Directional deep brain stimulation (DBS) lead is a major technological advance in the field. Currently, in the clinic, there are two 8-contact directional systems with FDA approval (Anderson et al., 2018). Considering that one of the largest restrictions of conventional DBS is the behavioral and cognitive side effects associated with the stimulation of non-motor functional areas in proximity to motor subthalamic nucleus (STN) in Parkinson’s disease (PD) (Pollo et al., 2014), the use of multi-contact directional leads might overcome these limitations. The main advantage of the segmented leads over traditional 4-contact leads is that stimulation current can be steered, and electrical field can be shaped with more asymmetric profiles (Pollo et al., 2014; Anderson et al., 2018; Rebelo et al., 2018). Promisingly, recent studies have reported that use of directional DBS leads might increase the therapeutic window (TW), decrease the therapeutic current strength needed to treat the majority of parkinsonian symptoms, and provide higher efficacy over non-directional approaches (Contarino et al., 2014; Pollo et al., 2014; Steigerwald et al., 2016; Dembek et al., 2017; Falconer et al., 2018; Rebelo et al., 2018; Tinkhauser et al., 2018).

While directional leads provide more flexible programming such that a given patient’s programming can be better customized for their unique symptoms, they also considerably increase the complexity of clinical programming, resulting in over 25,000 possible setting combinations (Anderson et al., 2018). In efforts to decrease the necessary time spent during programming, several computational models have been proposed to help predict the ideal stimulation settings once the lead has been placed (Kuncel and Grill, 2004; McIntyre et al., 2004; Brocker et al., 2017). Artificial neural networks have been shown to deliver quick, generalized computations concerning the volume of tissue activated from the lead (Chaturvedi et al., 2013). Another model utilizes a finite element model to compute the estimated neural activity in the targeted area (Anderson et al., 2018). Although these computational models have been shown to simplify programming of complex devices by computing optimal contact configurations, the success of these models depends heavily upon the accuracy of the lead placement.

Over the past decades, local field potentials (LFPs) have been used as an alternative, additive, or sole metric in many applications, such as in evaluation of medication (Brown et al., 2001; Priori et al., 2004; Kühn et al., 2006) and DBS effects (López-Azcárate et al., 2010; Eusebio et al., 2011; de Hemptinne et al., 2013, 2015), target localization and STN mapping (Zaidel et al., 2010; Wang et al., 2014; Yang et al., 2014; Telkes et al., 2016), patient programming/DBS efficacy (Brown and Williams, 2005; Ince et al., 2010; Priori et al., 2013; Thompson et al., 2014), and in many other applications. Specifically, excessive beta band (8–30 Hz) activity is widely reported in STN at rest (Levy et al., 2002; Kühn et al., 2008; Brittain and Brown, 2014) and it is further localized to dorsolateral part of the STN (Rodriguez-Oroz et al., 2001; Weinberger et al., 2006). Studies also demonstrated that dorsolateral section of the STN was closely related to tremor (Rodriguez-Oroz et al., 2001) and the dorsal part of the STN had different spectral patterns in patients with tremor dominant symptoms (Telkes et al., 2018).

However, functional use of LFPs in directional DBS systems has not been studied extensively. Little research has been done to understand whether certain directions of higher beta band power correlate to specific motor symptoms of PD. In this pilot study, we aim to investigate the possible relationship between directional LFPs in the STN and individual parkinsonian motor symptoms.

## Materials and Methods

### Patients and Surgery

Patients were recruited at Albany Medical Center, and written informed consent was obtained from all patients before participating in the study. The experimental protocol was approved by the Institutional Review Board at Albany Medical Center prior to beginning the study. LFPs were recorded intraoperatively in 8 patients (6 males, 2 females) diagnosed with PD who were scheduled to proceed with DBS surgery. Prior to surgery, patients were evaluated by a movement disorder specialist and Unified Parkinson Disease Rating Scale Motor scores (UPDRS-III) were collected 51.7 ± 21.9 (average ± SEM) days before the surgery. Primary symptoms of tremor (7 items, max score 28), rigidity (5 items, max score 20), limb-bradykinesia (8 items, max score 32), and axial-symptoms including postural instability and gait disturbances (5 items, max score 20) were documented.

Based on a standardized DBS protocol, directional DBS leads were uni-or-bilaterally placed (Infinity-6172, Abbott Laboratories, Illinois, United States) into the STN under local anesthesia. Surgical planning and targeting details have been previously described (Novak et al., 2011). Patients were asked to discontinue Parkinson’s medications 12 h before surgery and 2-or-3-track microelectrode recording (MER) single unit activities (SUA) were performed while patient was awake. Electrophysiological mapping started 10 mm above the estimated STN target with 0.5 mm steps and extended 3 mm below the target reducing to 0.3 mm steps within 6 mm of the target. The optimal track, the dorsal and ventral borders of the STN, and neighboring structures such as Zona incerta (ZI) and Substantia nigra (SNR) were determined based on standard practice (Wang et al., 2014; Yang et al., 2014; Telkes et al., 2016). The bottom contact of the directional lead was placed at the ventral border of the STN with the purpose of providing flexibility in steering the stimulation for the contacts sitting on the “sweet” spots. In addition to electrophysiological mapping and patient testing for intraoperative targeting, the position and the orientation of the lead were assessed using intraoperative fluoroscopy and postoperative computed tomography (CT) scan fused with the preoperative imaging. Finally, the electrode position was determined by referencing to the mid commissural point (MCP) using Brainlab software (Brainlab AG, Germany) (Supplementary Figure S1).

### LFP Recordings

For eight patients, directional LFPs were recorded from the hemisphere contralateral to the patient’s dominant motor symptoms. In one patient, signals were obtained from right and left hemisphere on different surgical dates, as the surgical procedures were staged for clinical reasons. Therefore, these recordings were counted as separate, enabling 9 individual directional lead recordings.

Data were acquired with Guideline4000LP Neuromodulation System (FHC Inc., Bowdoin, Maine) at 1 kHz sampling rate using a high-pass filter at 1 Hz and 16-bit A/D resolution. The LFPs referenced to cannula were obtained from all 8 channels in five recordings and 7 channels in three at rest over 1 m (Supplementary Table S1).

### Data Processing

Exported LFP data were analyzed using custom-developed Matlab (version R2014a; Mathworks, Natick, Massachusetts) scripts. The monopolar LFP data from all available contacts were high-pass filtered using a Butterworth filter with 2 Hz cutoff frequency to remove DC offset and 60 Hz notch filter to eliminate power line noise. In order to eliminate the common activity introduced by cannula, LFPs were re-referenced to common average during offline analysis (Supplementary Figure S2). Due to the differences in contact sizes and potentially in impedances (despite the measurements reported impedances in normal ranges), only the directional contacts were used to remove the mean activity.

**FIGURE 1:**
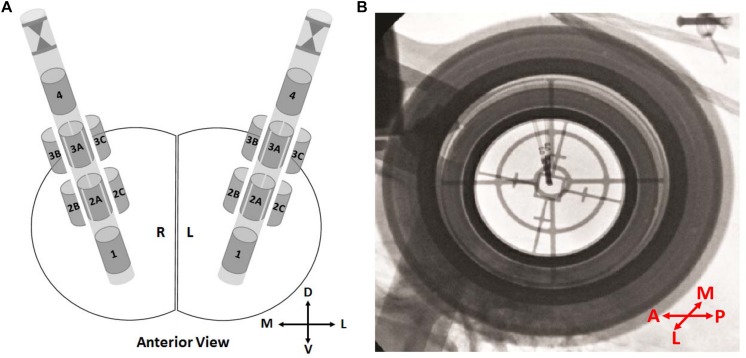
Directional leads and orientations. **(A)** Schematics showing the 8-contact directional leads in the right and the left hemisphere from the anterior view. Contacts 1 and 4 representing the ring contacts at bottom and top, respectively. The stereotactic marker and directional contacts 2A–3A indicating anterior. Depending on the implantation side (right/left), directional contacts 2B–3B and 2C–3C indicating lateral or medial. **(B)** An intraoperative fluoroscopy image from P1. The stereotactic marker facing anterior. In order to prevent any confusion, labels “2B–3B” and “2C–3C” were changed to “2M–3M” and/or “2L–3L” directions based on the hemisphere. D, dorsal; V, ventral; M, medial; L, lateral; A, anterior; P, posterior.

The directionality of the leads was determined in the operating room using fluoroscopy. The lead was placed so that the stereotactic marker was anterior, and data was interpreted appropriately. Since the labels on the lateral and medial directions (B/C) are opposite whether they are place in right or left, all the directions were corrected and labels were converted to true anterior, lateral, and medial directions (Figure 1A).

Spectral analysis was computed by a modified Welch periodogram method with robust statistics (Telkes et al., 2016). A 1,024-point fast Fourier transform was computed with a 1-second Hanning window and 50% shift and artifact-rejected time-frequency (TF) map was generated for each contact. In order to both visualize and quantify the spectral dynamics, power spectra were obtained from TF maps by keeping the directionality. From the power spectra, 3 features were extracted: (i) normalized beta band (13–20 Hz) power, (ii) normalized peak beta power (13–35 Hz), and (iii) peak beta frequency (13–35 Hz). Normalization was computed with respect to the total beta band power (13–35 Hz) and the features were converted to decibel scale for further analysis (Supplementary Note S1) (Tinkhauser et al., 2018). Due to small sample size, the directional features were compared by combining the depths (levels 2 and 3) in the same direction.

### Statistical Analysis

Statistical analyses were performed in MATLAB software version R2014a (The Mathworks, Inc., Natick, MA, United States). All data were presented as mean ± SEM. Normality and homogeneity of the data distribution were tested by Shapiro–Wilk test and Levene’s test, respectively. Due to the assumptions not being held, comparison of LFP features between directional contacts was carried out by the non-parametric Kruskal–Wallis test for unpaired samples. Correlations between UPDRS-III scores and LFP features were calculated using Spearman’s correlation analysis and correlation coefficient was represented by *r*. Multiple comparisons were corrected by the Bonferroni correction method. The significance threshold was set to 0.05 in all the statistical analyses.

## Results

Data were recorded from 9 hemisphere in 6 men and 2 women with a mean age of 67 ± 3.41 (mean age ± SEM; range: 49–77) years. The mean duration of the disease was 9.56 ± 0.88 years and mean preoperative improvement in UPDRS-III scores (medication OFF-to-ON) was 64.43 ± 4.97%. The average medication OFF scores for tremor, bradykinesia, rigidity, and axial symptoms were 6.13 ± 2.03, 16.13 ± 1.22, 5.50 ± 1.55, and 8.13 ± 1.16, respectively. The DBS lead positions were assessed both by intraoperative MER and postoperative CT scans. Postoperative assessment of the lead positions demonstrated that all leads were in the dorsolateral STN, except in P5. In P5, the position of the lead was found in caudal ZI and this patient was counted as outlier. The relative coordinates of the leads to the MCP were reported in Supplementary Table S1 and Supplementary Figure S1D.

### Directional Patterns of Resting State LFPs

Orientation of the directional leads with stereotactic marker facing anterior was shown in Figure 1A. The most dorsal and ventral contacts were represented as contacts 1 and 4, respectively. Figure 1B demonstrates the intraoperative fluoroscopy image in a representative patient. Image provides two type of information: (i) orientation of the lead based on the marker, (ii) position of the lead based on the tip. As seen in Figure 1B, marker is clearly facing anterior direction. The position and the orientation of the lead were confirmed in all patients.

**FIGURE 2:**
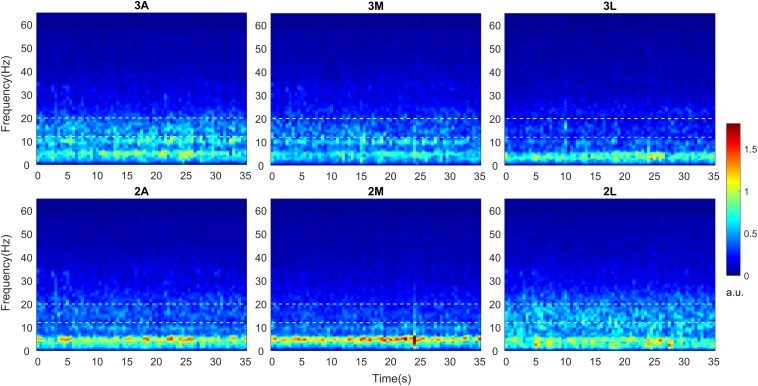
Time-frequency analysis of directional LFPs. Intraoperative LFPs were obtained in awake patients at rest with stimulation OFF (baseline). Time-frequency maps were computed for all available DBS contacts after LFPs were re-referenced to common average. Time-frequency maps averaged across 9 hemispheres indicating spectro-temporal dynamics of directional LFPs. The x axis represents time in seconds and the y axis represents frequency in Hz. The white dashed lines represent the borders of low beta band (13–20 Hz). The color bar indicates the energy of the signal in arbitrary unit (a.u.). A, Anterior; M, Medial; L, Lateral.

The grand averages of time-varying spectral changes for baseline LFPs were demonstrated in Figure 2. The duration of the recordings was 52.85 ± 2.59 s in average. The TF maps for 35 s period were shown in anterior, medial, and lateral directions at two depths. Visual inspection of the maps indicated distinct beta band (13–20) activity, in which borders were marked with white dashed lines. The dorsoanterior (3A) and ventrolateral (2L) contacts showed stronger beta activity while the smallest beta activity was located to ventromedial (2M) contact. The excessive activity in theta (4–8 Hz)-alpha bands (8–12 Hz) also indicated spatially distinct distributions across directions while no visible activity was noted in gamma (35–70 Hz) range.

**FIGURE 3:**
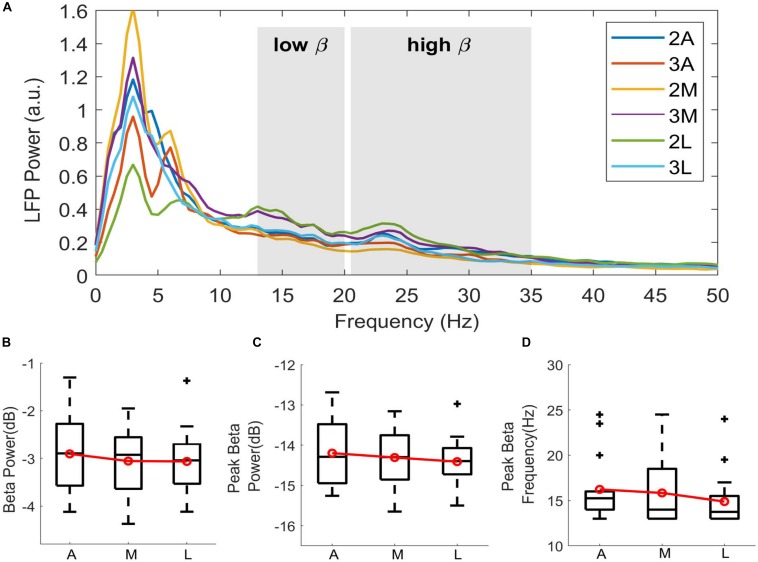
Directional distribution of spectral features. **(A)** Spectra showing the LFP power distribution of directional contacts computed from the time-frequency maps by averaging over time in a representative patient (P6). Shahed areas indicating low beta (13–20 Hz) and high beta (20–35 Hz) bands. Despite the clear peak activity in lateral contact 2L (green), not every contact demonstrates clear beta peak in low beta. Boxplots showing the distribution of **(B)** normalized beta power in dB scale, **(C)** normalized peak power in beta range in dB scale, and **(D)** peak beta frequency in Hz. Contacts on the second and the third levels were directionally combined to enhance the emphasis on the directionality. A, Anterior; M, Medial; L, Lateral. Red lines indicating group mean. + indicating outliers, *p* > 0.05.

In order to inspect the spectral changes individually and extract the subband features, the power spectra per hemisphere were computed from the TF maps by averaging over time. Figure 3A demonstrates the spectral power dynamics of directional contacts in a representative patient. Ventrolateral contact (2L) shows the highest beta activity, followed by dorsomedial (3M) contact. Interestingly, these two contacts showed clear peaks in low (13–20 Hz) and high (20–35) beta bands while the rest of the contacts did not show any clear peak in low beta range. Similar to the average TF maps, the smallest beta activity was localized to ventromedial (2M) contact in the patient. We observed different patterns across patients.

To further investigate these patterns, low-beta power was extracted and normalized to total beta power. Additionally, peak power and its corresponding frequency were computed in wide beta range (13–35 Hz). Figures 3B–D shows the box-and-whisker plots of log-transformed normalized beta band features and peak frequencies in 3 directions. Due to small sample size, the dorsal and ventral contacts in the same directions were combined (Supplementary Figure S4). Distributions showed higher subband and peak power in the anterior direction. Median value of the beta power was –2.895 in anterior, –2.925 in medial, and –3.042 in lateral. Median value of the peak power in beta range was comparable across the contacts indicating –14.289 in anterior, –14.310 in medial, and –14.395 in lateral. Peak frequency distribution indicated that median peak frequency was 15.24 Hz in anterior, 14 Hz in medial, and 13.75 Hz in lateral directions. Despite the trend between directions, statistical analysis did not show any significant difference with beta band power [χ^2^_(2)_ = 0.52, *p* = 0.769], peak power [χ^2^_(2)_ = 0.52, *p* = 0.768], or peak frequency [χ^2^_(2)_ = 2.89, *p* = 0.235].

### Motor Symptoms Comparative Analysis

**FIGURE 4:**
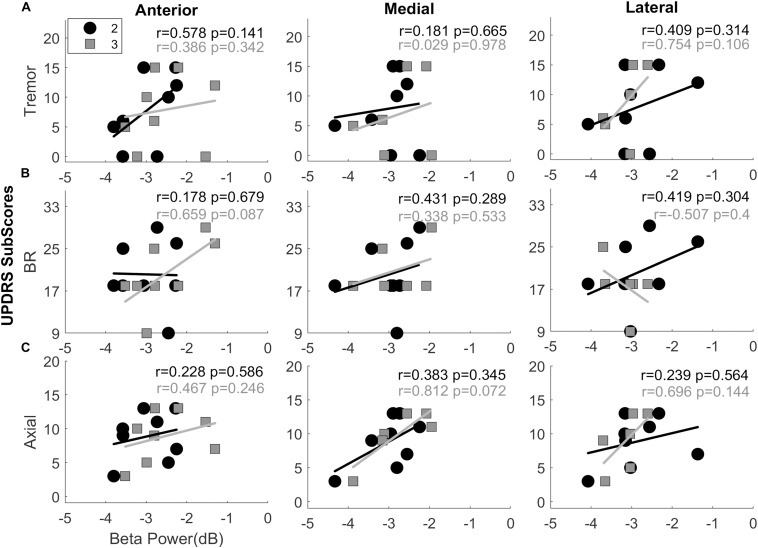
Interactions between UPDRS sub-scores and normalized beta band power. Scatter plots showing the interactions between beta band power vs. Tremor **(A)**, Bradykinesia and Rigidity **(B)**, and Axial scores **(C)** in anterior, medial, and lateral directions. The second (black circles) and third level (gray squares) contacts were demonstrated on the same plot. The trends were found to be mostly positive with tremor scores and more uniform with the axial scores. All directions and all levels indicate positive interaction. Combination of bradykinesia-rigidity (BR), on the other hand, were mixed. Top-anterior (level-3) directional contact shows the clearest positive trend between beta band power and BR scores. It might be due to orientation of the lead facing anterior with the assumption that it is in motor region of the STN. Spearmen correlation analysis did not show significant correlation between beta power and sub-scores in any direction. *r*, Spearman’s correlation coefficient.

The average TF maps demonstrated different spectral dynamics across directional contacts. In order to study whether these differences are symptom-related, we assessed the correlation between normalized beta power and UPDRS-III sub-scores: (i) tremor, (ii) bradykinesia/rigidity, and (iii) axial scores, respectively. Results of the Spearman correlation analysis did not reach to statistical significance in any directions; however, it demonstrated positive trends with bradykinesia/rigidity in dorsoanterior direction (*r* = 0.659, *p* = 0.087; Figure 4B) and with axial scores in the dorsomedial direction (*r* = 0.812, *p* = 0.072; Figure 4C). Trends were generally positive with tremor and axial scores in all directions as well as across the depths. The higher variance was observed with the bradykinesia/rigidity scores in anterior and lateral directions between dorsal and ventral contacts. Is this because bradykinesia/rigidity, which are usually found to be correlated with beta power in PD (Kühn et al., 2006, 2009; Weinberger et al., 2006), are more specific to direction? This needs to be further investigated within a larger population.

## Discussion

The present study showing the LFP dynamics recorded from FDA-approved 8-contact DBS leads with the two inner most trifurcated contacts (1.27 mm diameter, 1.15 mm contact width; model # 6172, Abbott Laboratories, IL, United States) is one of the few studies in literature (Fernández-García et al., 2017; Tinkhauser et al., 2018). Here with our two trifurcated directional leads, in this pilot study, we examined the anterior, medial and lateral oscillatory dynamics of the STN and their relationship to motor symptoms of PD. LFP data collected intraoperatively showed that the normalized beta band power observed in the STN was higher in anterior direction and had positive trends with bradykinesia/rigidity in dorsoanterior and with axial scores in the dorsomedial directions.

Functional use of LFPs, which represent the aggregated synaptic potentials of a population of neurons (Buzsáki et al., 2012), have been shown by many groups in basal ganglia mapping and STN localization (Chen et al., 2006; Gross et al., 2006; Telkes et al., 2014; Wang et al., 2014), assessment of clinical improvement by medication or DBS (Foffani et al., 2003; Ray et al., 2008; Özkurt et al., 2011; de Hemptinne et al., 2013; Ozturk et al., 2020), and selection of programming contacts (Ince et al., 2010; Zaidel et al., 2010; Connolly et al., 2015). More specifically, increased beta band (13–35 Hz) oscillatory activity in the STN was correlated to pathological state in PD. The suppression of these oscillations by dopaminergic medication improved bradykinesia and rigidity in PD patients (Kühn et al., 2006, 2009). When the location of these oscillations and their correlation to motor symptoms were considered, one study showed beta oscillating units localized to the very dorsal border of the STN which were strongly correlated with rigidity while the sub-beta power was significantly further from the STN entrance and strongly correlated with axial scores (Sharott et al., 2014). Similarly, higher beta power and significant beta-slow high frequency coupling was localized to dorsal STN in PD patients with dominant tremor while more ventral depths indicated significantly stronger beta-fast high frequency coupling in patients with dominant axial symptoms (Telkes et al., 2018). These findings suggest that there are cells with distinct firing characteristics in the territories of STN which individually correlate with the cardinal motor symptoms of PD. We speculate that our findings with differences in directional distribution of spectral features and their interactions to motor symptoms of PD might be an indicator of these focal neural groups.

One of the major challenges of using segmented leads is that programming might require a huge amount of time given that there are 8 contacts per hemisphere and numerous combinations of pulse width (20–500 μs), frequency (2–240 Hz), and current (0–12.75 mA). Even though tremor response to STN DBS usually occurs immediately (Krack et al., 2002), symptoms such as bradykinesia and rigidity take longer to be suppressed by DBS, and they return more slowly (Volkmann et al., 2002; Temperli et al., 2003). Another factor limiting parameter selection is that there is no single parameter setting that works for everyone with the same effectiveness. To this end, research on directional LFPs has shown that increased beta band power obtained from directional contacts can predict the patient’s optimal contacts and the widest therapeutic window (Bour et al., 2015; Tinkhauser et al., 2018) given that beta band activity has been shown to be higher in the contacts which are closer to the source (Ince et al., 2010; Tinkhauser et al., 2018).

Connectivity analysis demonstrated that the sensorimotor region of STN with the highest beta band power, located in the dorsolateral region, is associated more with the primary motor cortices (Accolla et al., 2016; Horn et al., 2017). Specifically, Horn and colleagues showed that largest beta activity was located to dorsolateral part of the STN with higher connectivity to motor cortex whereas the highest alpha activity was located to ventromedial part, which might be within the associative part of STN, indicating strong connectivity to premotor/frontal cortical areas (Horn et al., 2017). In our time-frequency maps, we observed stronger beta activity localized to the anterior direction. When the surgical approach was considered where bottom contact of the lead is placed at the ventral border of the STN to provide flexibility of steering to the directional contacts, anterior contacts- confirmed with intraoperative fluoroscopy- might be just in the motor region of the STN. Given that stimulation of the dorsolateral region of STN has previously been shown to improve PD symptoms (Zaidel et al., 2010), positive trends between spectral features and the bradykinesia/rigidity scores in anterior direction might be an indicator of “sweet” spot in the motor STN.

There are several limitations of this study. Such limitations include small number of subjects and intraoperative time constraints. Having the LFPs recorded for a longer period of time would enhance the signal-to-noise ratio and be more representative. Additionally, due to hardware limitations, we could not obtain high frequency oscillations. Given that recent studies have shown the functional role of coupling between beta band and high frequency oscillations in PD, it would provide a broader insight into spatio-spectral patterns and pathophysiological mechanisms in PD. During the LFP recordings, we did not take into account whether the patient had tremor, if so, how severe the tremor was. It is essential to conduct studies with a larger patient population to evaluate the clinical utility of using LFP data to guide DBS programming and fully establish the limitations of this approach.

## Conclusion

The purpose of this pilot study was to investigate the spectral differences of intraoperatively recorded LFPs between directional contacts and their correlations to distinct motor symptoms of PD. Given the time limitations associated with programming of directional devices, further investigation of these findings in a larger cohort and within a wider frequency range with non-linear interactions might serve as an initial metric for optimal clinical contacts. LFPs carry pathological signatures of PD and may provide a functional use to predict optimal stimulation parameters in future.

## Data Availability Statement

The datasets generated for this study are available on request to the corresponding author.

## Ethics Statement

The studies involving human participants were reviewed and approved by Institutional Review Board at Albany Medical Center. The patients/participants provided their written informed consent to participate in this study.

## Author Contributions

IT and JP designed the study, collected the neural data, and performed the data analyses. IT, JP, and SS wrote the manuscript. JP and VS performed the surgeries, contributed to data collection, and interpretation of the results. JD and OA conducted patient testing during and after the surgery and contributed to interpretation of the results. SS and NR provided the patient’s demographics and clinical information. MS provided intraoperative and postoperative radiographic images and contributed to interpretation of the results. All authors reviewed the manuscript and approved the final version of the manuscript.

## Conflict of Interest

JP is a consultant for Boston Scientific, Nevro, Jazz Pharmaceuticals and Abbott and receives grant support from Medtronic, Boston Scientific, Abbott, Nevro, Jazz Pharmaceuticals, GE Global Research, NIH 2R01CA166379-06, and NIH U44NS115111. She is the medical advisor for Aim Medical Robotics and Karuna and has stock equity. The remaining authors declare that the research was conducted in the absence of any commercial or financial relationships that could be construed as a potential conflict of interest.
