# Supplementary material for: Functional Use of Directional Local Field Potentials in the Subthalamic Nucleus Deep Brain Stimulation
Source: Front Hum Neurosci. 2020 Apr 28;14:145. doi: 10.3389/fnhum.2020.00145 (PMC7198898; doi:10.3389/fnhum.2020.00145)
Supplement: Supplementary file 1 [file Presentation_1.pdf]

Supplementary Material

Supplementary Table S1. Demographics and Clinical Information											
ID	STN side	Lead Coordinates[a]			Disease duration (yr)	UPDRS[b] Sub-scores				UPDRS Total	LFP channels
		X	Y	Z		Tr	Ri	Br	Ax	OFF/ON	
1	Right	12.74	1.79	4.25	12	0	9	20	11	23/45	7
2	Right	11.23	3.40	3.40	7.5	1	12	18	7	24/44	7
3	Left	11.79	1.48	2.99	12	10	0	9	5	3/25	7
4-5	Left	9.92	2.01	4.8	12	15	2	16	13	15/53	8
	Right	11.64	5.28	2.09							8
6	Left	10.52	2.47	3.83	5	0	1	17	10	10/35	8
7	Left	12.24	0.21	3.79	10	12	7	19	7	28/20	5
8	Right	10.92	2.99	4.34	9	5	4	14	3	9/29	8
9	Left	8.37	2.01	4.57	9	6	9	16	9	13/43	8

a: Lead coordinates indicating tip of the electrode relative to mid commissural point (MCP). XYZ: lateral, posterior, inferior, respectively. b: Unified Parkinson Disease Rating Scale-III sub-scores obtained during medication OFF. Tr: Tremor. Ri: Rigidity. Br: Bradykinesia. Ax: Axial scores.

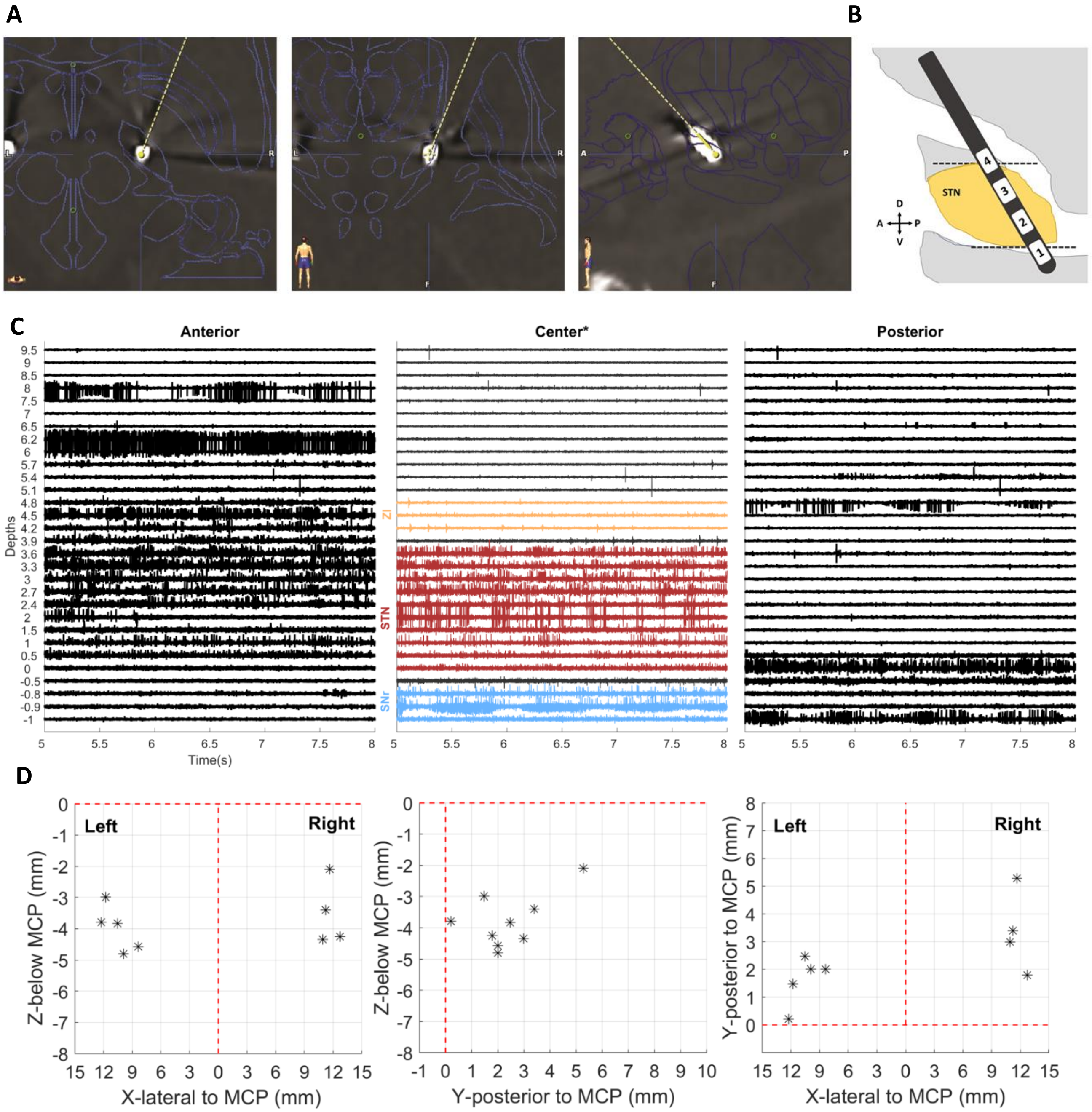

Supplementary Figure S1. Localization of DBS lead. (A) The postoperative CT overlay on atlas in 3 planes in P3. From left to right: axial, coronal, and sagittal. **(B)** A representative schematic of DBS lead placed in the STN (sagittal view). Black dashed lines indicating dorsal and ventral STN borders determined by microelectrode recordings and imaging. **(C)** Three-track microelectrode (MER) single-unit activity (SUA) recording in a representative patient. X-axis indicating time in seconds, y-axis indicating depth in mm. The center track marked with star showing the selected track for the DBS lead implantation. Colors representing different structures. Yellow: Zona incerta (ZI). Red: Subthalamic nucleus (STN). Blue: Substantia nigra (SNr). **(D)** Distribution of lead coordinates relative to mid commissural point (MCP) in 3 planes: coronal, sagittal, and axial, from left to right respectively.

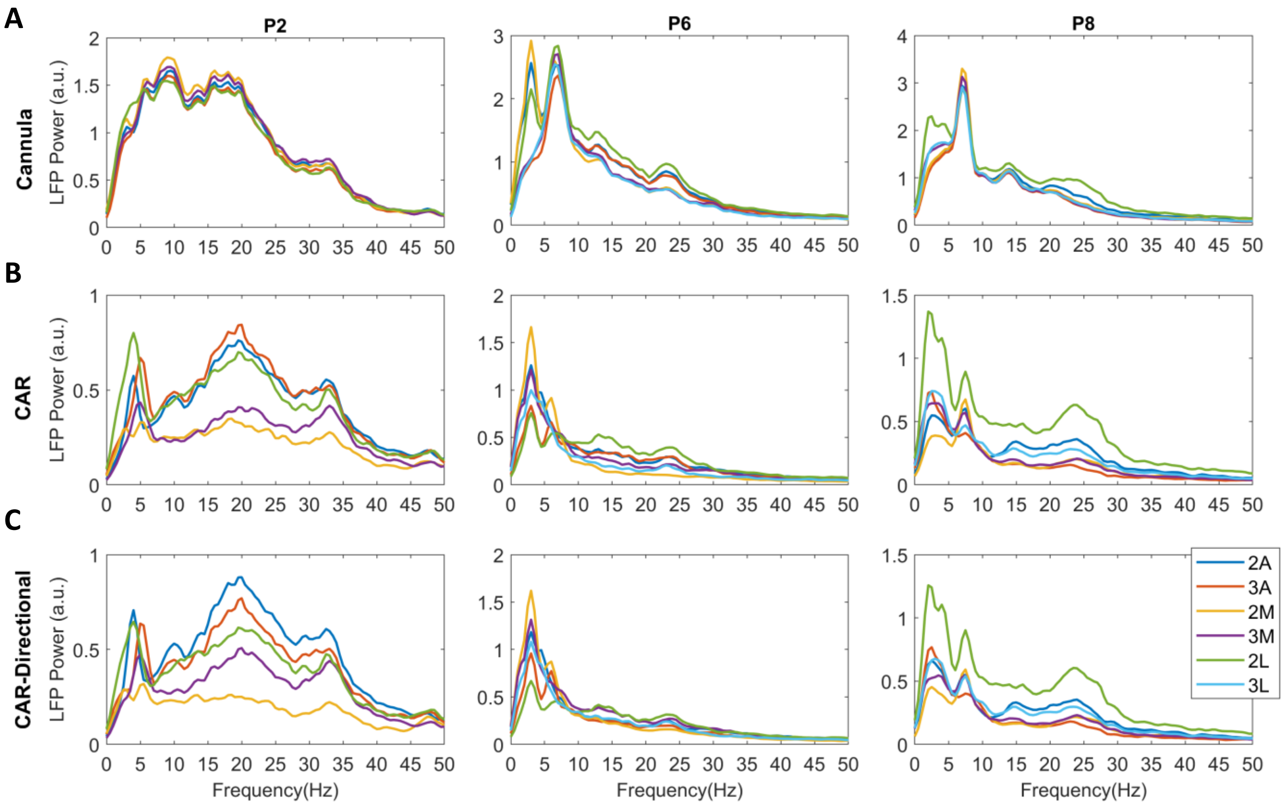

Supplementary Figure S2. Comparison of references. Plots showing the time-averaged power spectra in 3 representative subjects with different references. **(A)** LFPs were referenced to cannula during the recordings**. (B)** LFPs were re-referenced to common average (CAR) during offline analysis. **(C)** CAR was computed only across directional contacts. Re-referencing eliminates the common activity across the contacts compared to using cannula as reference and enhances the focal LFP dynamics. Comparison between global CAR and directional CAR in P2 shows slightly different peak power distribution between anterior contacts (2A vs 3A). It might be because of a strong activity in the bottom ring contact. Due to differences in contact sizes and potentially in impedances, despite the measurements reported impedances in normal ranges, it would be fair to use only the directional contacts to remove the mean activity. Therefore, spectral features were computed only for the directional contacts after removal of common activity as indicated. Each color representing a directional DBS contact.

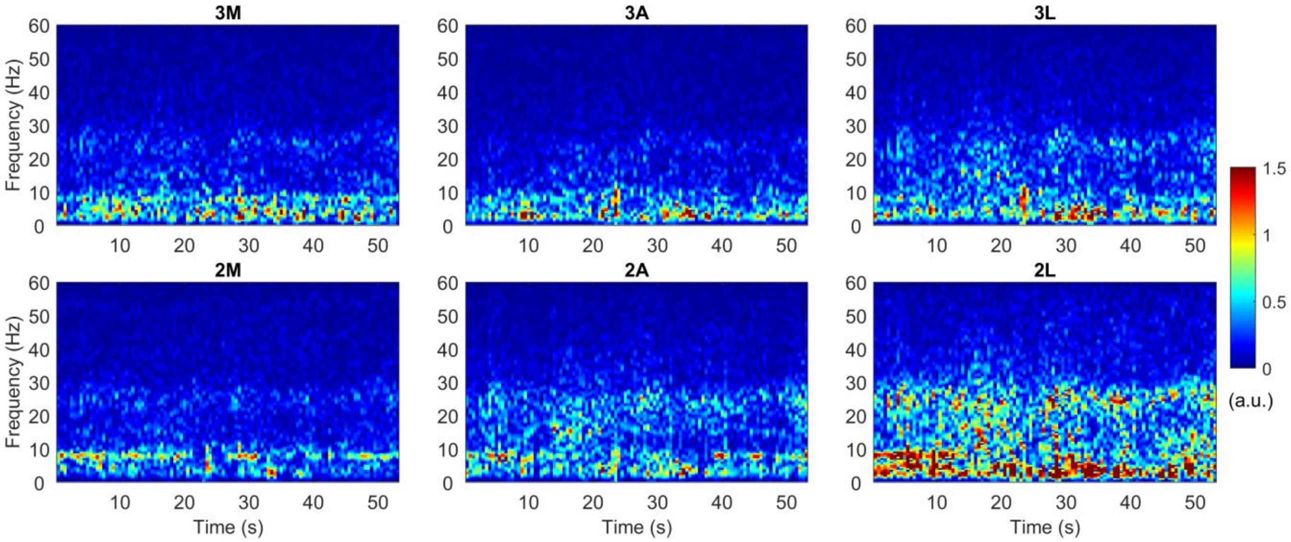

Supplementary Figure S3. Time-frequency dynamics in directional contacts. Intraoperative LFPs were obtained in awake patients at rest with stimulation OFF (baseline). The average duration of the baseline recordings included in analysis is 52.85 ± 2.59 s (mean ± standard error of mean). Time-frequency maps were computed for all available DBS contacts after LFPs were re-referenced to common average. Maps showing the distribution of LFP power in the directional contacts up to 60Hz and over time in a representative patient (P8). Color bar indicating LFP power in arbitrary unit (a.u.).

Supplementary Note S1. Equations showing the computation of features to be used in groups analysis. Subband power of low beta (13-20Hz) is normalized to total power of the entire beta band (13-35Hz) as shown in Eqn-1. Furthermore, peak power was computed in beta band (13-35Hz) range and normalized to total beta band power as shown in Eqn-2. Frequency corresponding to peak beta powers were also computed.

$$Power_{normalized} = 10 \times log_{10} \left(\frac{Power_{beta\_low}}{Power_{beta}}\right), \quad (1)$$

$$PeakPower_{normalized} = 10 \times log_{10} \left(\frac{PeakPower_{beta}}{Power_{beta}}\right), \quad (2)$$

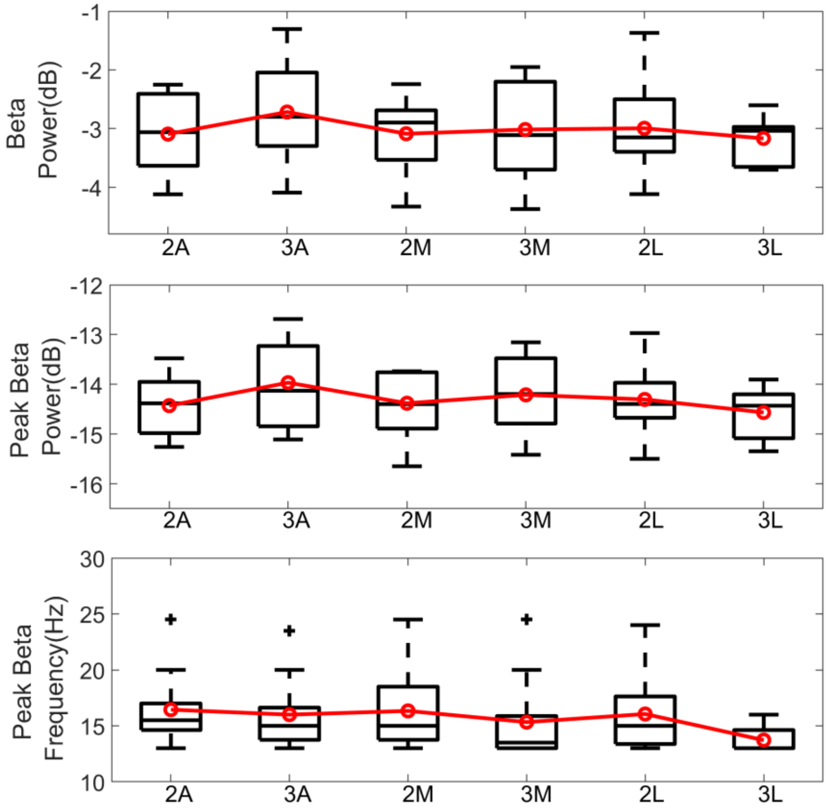

Supplementary Figure S4. Directional distribution of spectral features. (A) normalized beta power (13-20Hz) in dB scale, **(B)** normalized peak beta power (13-35Hz) in dB scale, and **(C)** peak beta frequency (13-35Hz) in Hz. Contacts on the second and the third levels representing ventral and dorsal directional contacts, respectively. A: Anterior. M: Medial. L: Lateral. Red lines indicating group mean. + indicating outliers. Kruskal–Wallis analysis did not show any significant difference. p>0.05

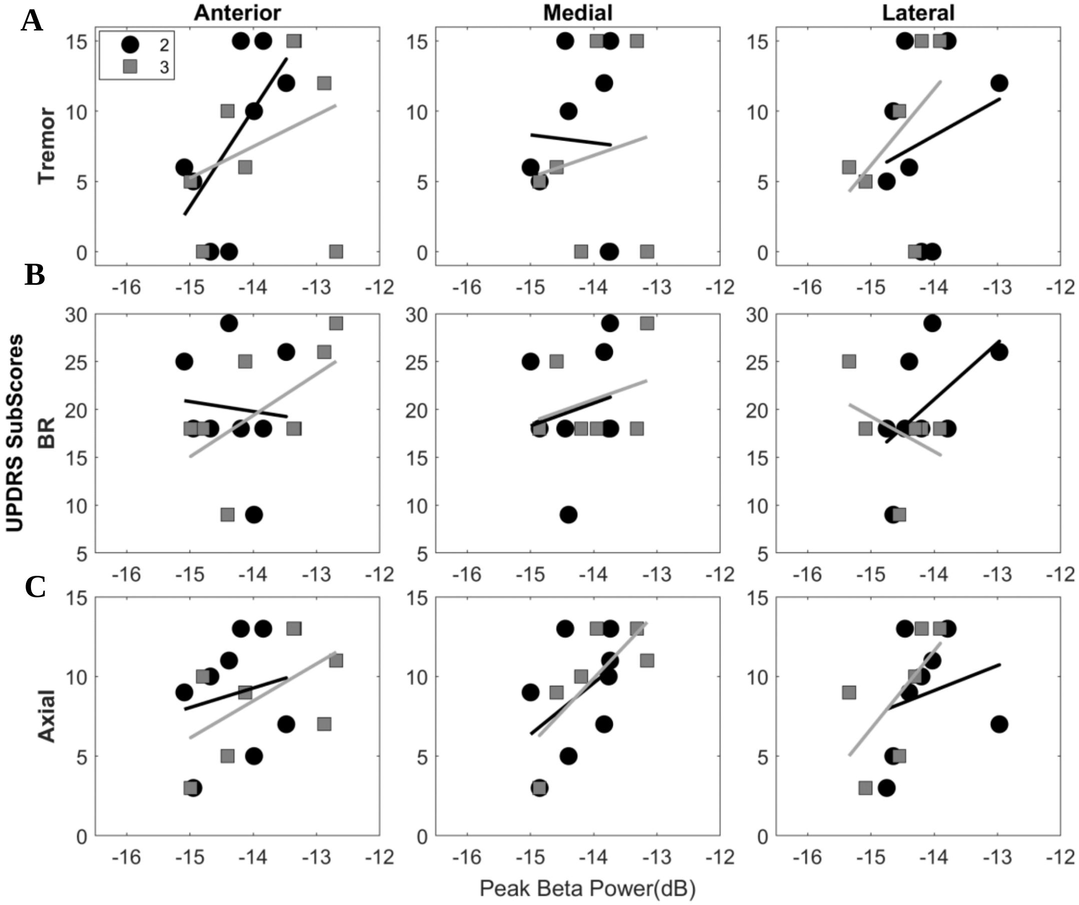

Supplementary Figure S5. Interactions between UPDRS sub-scores and normalized peak power. Scatter plots showing the interactions between peak power in beta band vs Tremor **(A),** Bradykinesia and Rigidity (BR) **(B),** and Axial scores **(C)** in anterior, medial, and lateral directions. Distribution of normalized peak power are highly similar to normalized subband power. Therefore, correlation analysis shows similar interaction maps. The trends between peak beta power and BR scores in anterior was r=0.685, p=0.065; with axial scores in the dorsomedial direction was r=0.812, p=0.072.
